# Supplementary material for: Extreme melt season ice layers reduce firn permeability across Greenland
Source: Nat Commun. 2021 Apr 20;12:2336. doi: 10.1038/s41467-021-22656-5 (PMC8058076; doi:10.1038/s41467-021-22656-5)
Supplement: Supplementary file 1 — Supplementary Information [file 41467_2021_22656_MOESM1_ESM.pdf]

Supplementary Information for

Extreme Melt Season Ice Layers Reduce Firn Permeability Across Greenland

Riley Culberg[1*], Dustin M. Schroeder[1,2], Winnie Chu[3]

[1] Department of Electrical Engineering, Stanford University, Stanford, CA, USA
[2] Department of Geophysics, Stanford University, Stanford, CA, USA
[3] School of Earth and Atmospheric Sciences, Georgia Institute of Technology, Atlanta, GA, USA

[*] Corresponding author: culberg@stanford.edu

This PDF file contains:

Supplementary Figures 1-5
Supplementary Tables 1-2
Supplementary Methods

Supplementary Figures

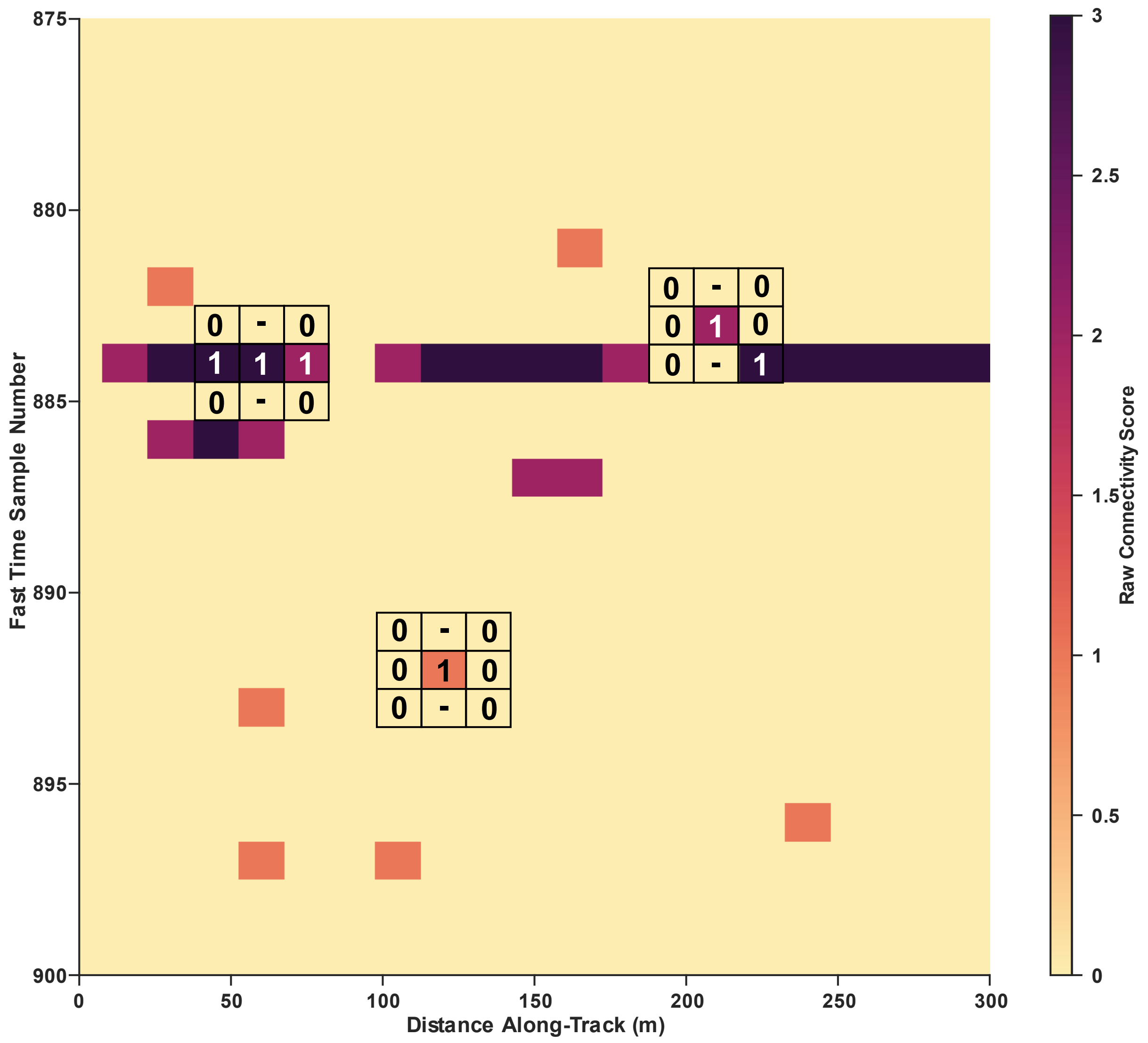

Supplementary Figure 1. Close-up image of the lateral layer connectivity method at the native horizontal trace spacing of the processed Accumulation Radar data (~ 15 m). The connectivity of each layer detection (orange or purple pixels in the image) is scored based on the lateral adjacency of other layer detection within a 3x3 kernel (examples shown in the black boxes).

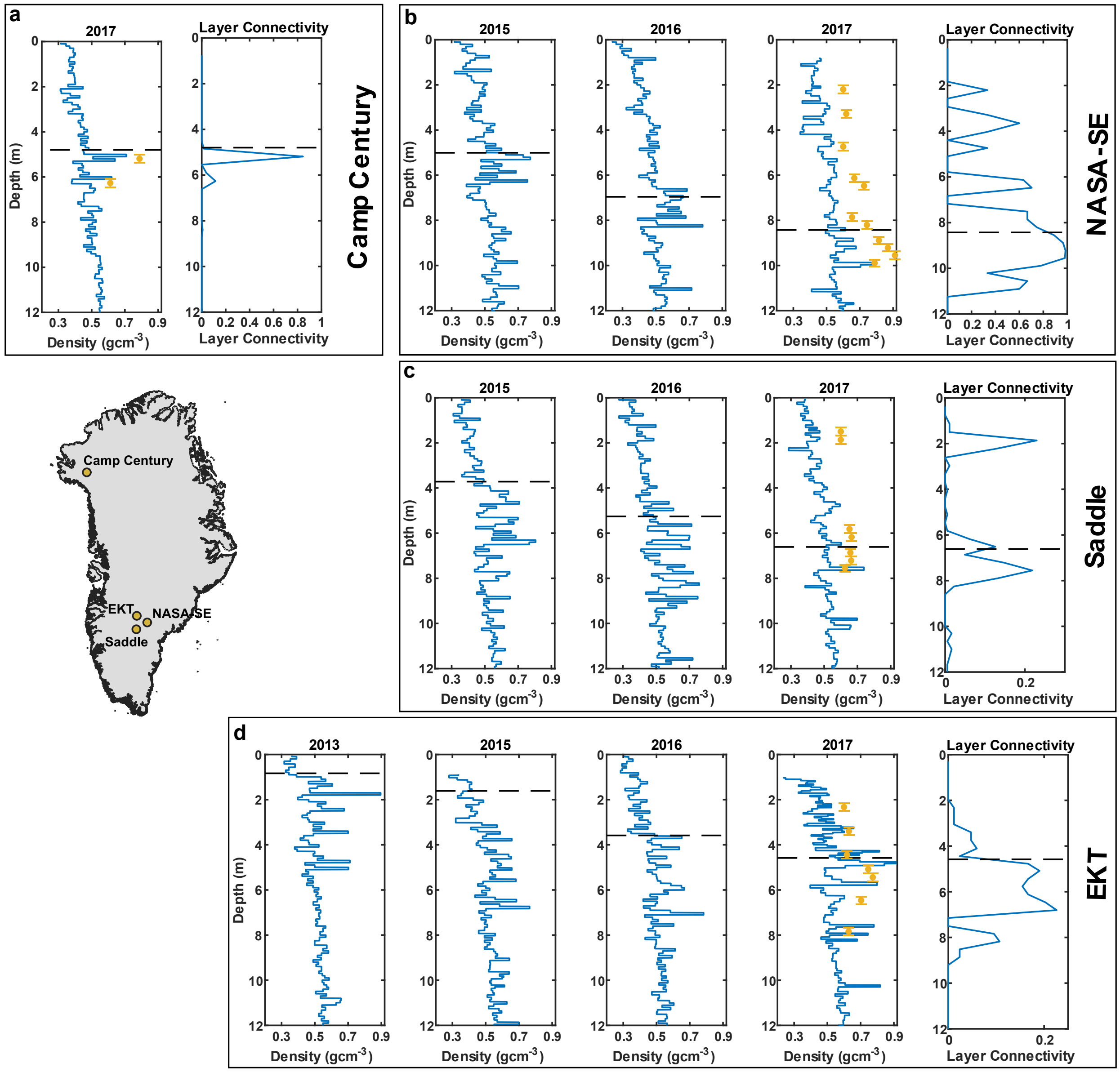

Supplementary Figure 2. Comparison of radar-derived melt layer depths and densities with firn cores observations collected by the Camp Century survey[1] and FirnCover[2–4] project (locations show on inset map in yellow). Density profiles are shown in blue, with the exception of the final plot in each section where the blue curve shows radar-derived layer connectivity. Dashed black lines show the modeled depth of the 2012 end-of-summer surface at the time of collection. Yellow dots indicate the depth and density of ice layers detected in the Accumulation Radar data as part of this study. a) Camp Century site in northwest Greenland. b) NASA-SE site in southeastern Greenland. c) Saddle site in the southern saddle. d) EKT site in southwestern Greenland.

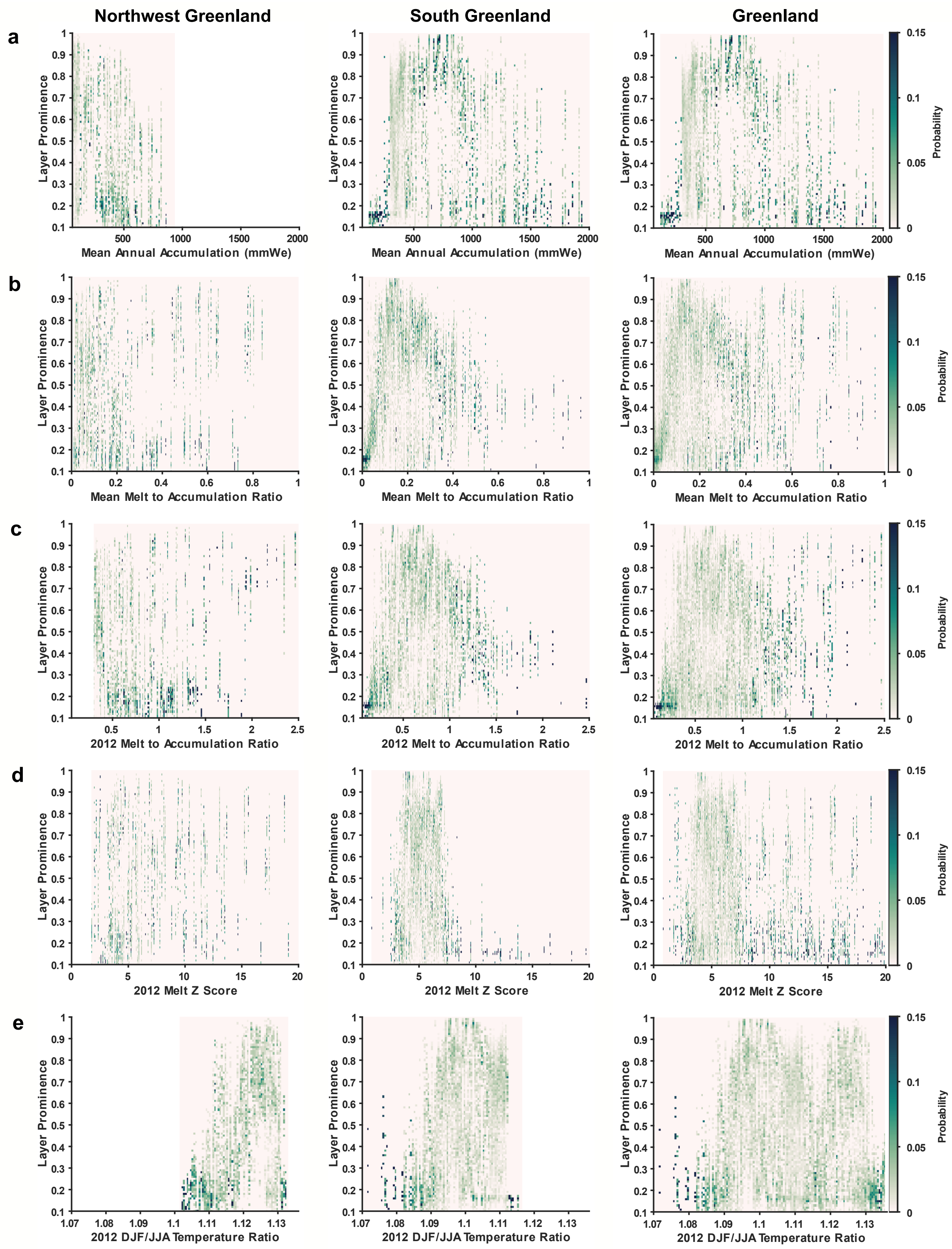

Supplementary Figure 3. Two-dimensional histograms showing the probability of a given radar-derived layer prominence occurring for a given climate condition for variables not shown in Fig

3. Darker colors indicated higher probabilities. Greenland regions are delineated in Figure 2c. a) Mean annual accumulation for the period 1980-2011 as simulated by MARv3.5.2[5]. b) Ratio of mean annual surface melt production to mean annual accumulation for the period 1980-2011. c) 2012 melt to accumulation ratio. d) 2012 normalized surface melt anomalies relative to 1980-2011. e) Ratio of the mean 2012 summer (JJA) surface temperature to mean 2011/2012 winter (DJF) surface temperature.

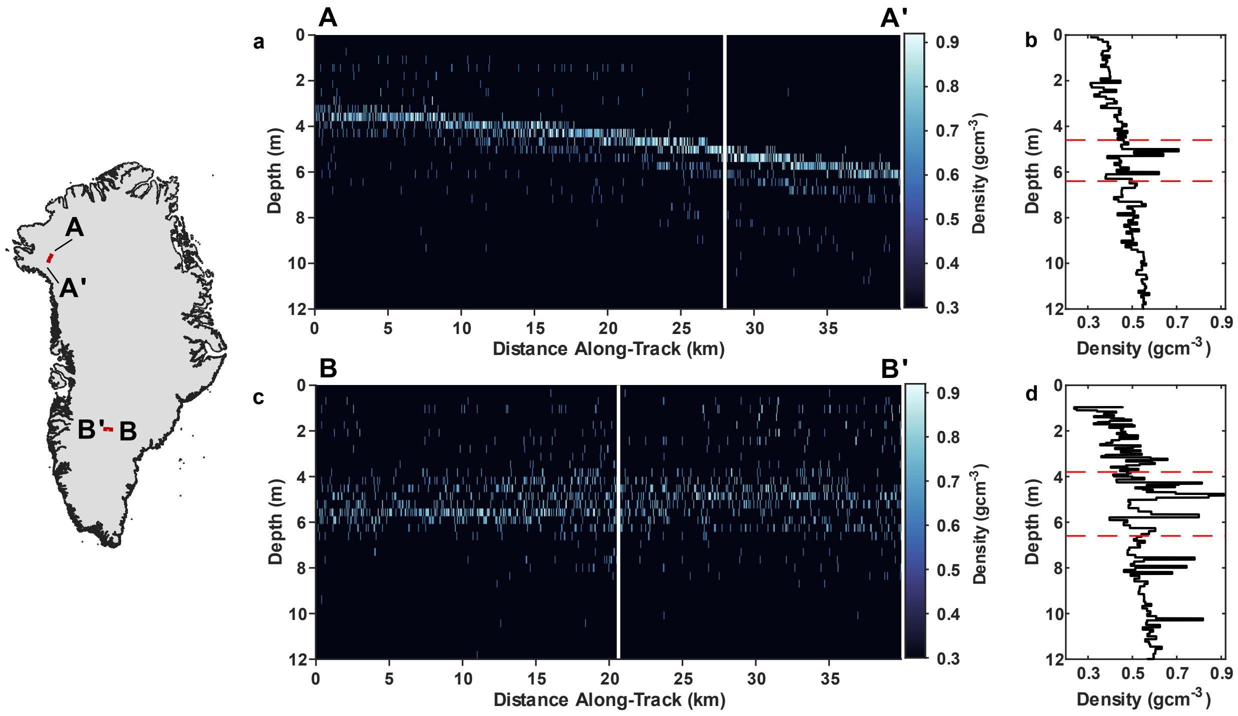

Supplementary Figure 4. Radar reflection character of the 2012 melt layer. The white lines in a) and c) show the point of closest approach to the firn cores shown in b) and d). The red dashed lines in b) and d) show the bounding depths of the radar-derived melt layer. Image colors in a) and c) represent the estimate density of detected ice layers, with brighter colors indicating higher density. The melt layer in northwest Greenland (a) is typically a single, well-defined reflector, consistent with the isolated density peaks in the Camp Century firn core[1] shown in b). The melt layer in the south (c) is a conglomeration of many bright reflectors, consistent with the thicker and more variable package of high-density peaks between 4-6 m deep in an EKT firn core[3,4] collected in May 2017 (d).

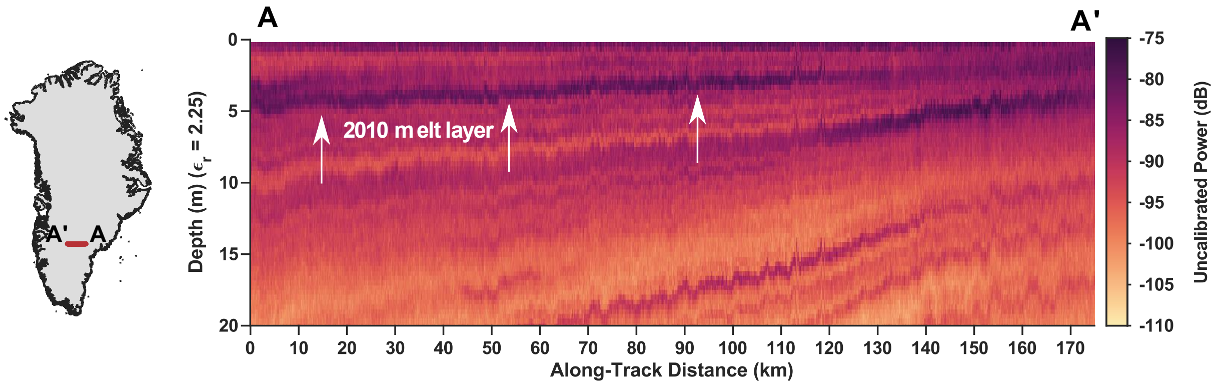

Supplementary Figure 5. Radargram showing the 2010 melt layer. The image colors represent the uncalibrated received radar power. Darker colors indicated higher power returns, consistent with large internal density contrasts within the firn. This data was collected in April 2012 by the CReSIS Accumulation Radar in the same location as Transect B in Fig 4.

Supplementary Tables

Supplementary Table 1. Distance correlation coefficients between radar-inferred layer prominence and MAR-modeled climate variables

Climate Metric	Northwest Greenland	South Greenland	All Greenland
Ratio of Latent Heat to Cold Content	0.54	0.4	0.28
STD of Daily Melt Rates (Melt Variability)	0.36	0.45	0.39
Mean Annual Accumulation	0.38	0.26	0.15
Mean Annual Melt to Accumulation Ratio	0.18	0.31	0.28
2012 Melt to Accumulation Ratio	0.36	0.31	0.25
Normalized 2012 Melt Anomalies	0.20	0.25	0.33
Ratio of JJA to DJF Mean Surface Temperatures	0.54	0.28	0.14

Supplementary Table 2. Modeled depth of the 2012 melt layer within the Helheim Firn Aquifer

Densification Scheme	Modeled 2012 Melt Layer Depth
Herron and Langway Dynamic	7.28 m
CROCUS	7.53 m
Arthern, 2010 Transient	8.09 m
Barnola, 1991	7.3 m
Kuipers-Munneke, 2015	8.14 m

Supplementary Methods

S1. Estimated Layer Density Contrast and Thickness

Monostatic ice penetrating radar systems are largely sensitive to sharp vertical gradients in the dielectric constant of the material they are sounding. In the upper few hundred meters of an ice sheet accumulation zone, these gradients are due almost entirely to variations in firn density[6,7]. We assume a simple empirical relationship between dry density and electrical permittivity of the form in equation 1 (ref 8), where ϵ' is the real part of the complex permittivity and ρ is the density in gcm^{-3}.

$$\epsilon' = (1 + 0.845\rho)^2 \tag{1}$$

In the dry snow zone, snow density varies between seasons and accumulation events[9], forming centimeter-thick stratigraphic layers which reflect radar waves and which are often continuous across hundreds of kilometers[10,11]. Ice layers which are relatively flat and continuous across the radar system first Fresnel zone will produce a similar effect. The strength of the radar reflections depends on the real part of the permittivity (ϵ'), and therefore density, difference between layers.

$$R = \left|\frac{\sqrt{\epsilon'_1} - \sqrt{\epsilon_2{}'}}{\sqrt{\epsilon'_1} + \sqrt{\epsilon'_2}}\right|^2 \tag{2}$$

The density contrast between air and the ice sheet surface is on the order of 0.3 gcm^{-3} for typical Greenland surface densities[12]. Therefore, for a subsurface reflector to be as bright or brighter than the surface return, there must exist some subsurface layer whose density is about 0.3 gcm^{-3} greater than or less than the density of the surrounding firn.

We assess that the 2012 melt layer is generally less than 0.3 m thick because it is characterized by a bright reflector rather than a continuous zone of low reflectivity. If the melt layer thickness exceeded the range resolution of the radar system, we would expect to see a bright reflection at the top boundary of the layer, a zone of low reflectivity within the layer where the ice is homogenous and minimal scattering occurs, followed by a bright reflection at the lower boundary of the layer. This would be similar to the scattering character of the ice slabs as described in MacFerrin, et al (2019). As we generally see single bright reflectors, this suggests that the top and bottom of the melt layer are not separately resolvable, meaning that the total thickness must be less than the range resolution of the radar system. In the case of the Accumulation Radar used in this study, the theoretical range resolution is about 0.3 m.

S2. Expanded Radar Processing Methods

Here we expand on our surface tracking and calibration methods.

Surface Tracking

Almost all of our analysis relies on a good record of the surface return in the radar data. While initial surface picks are provided with the processed CReSIS data, there are many places where the tracking is not sufficiently precise or includes major errors such as tracking transmitter feedthrough as the surface. In the 2013 data set in particular, there are also many locations where the 2012 melt layer is misidentified as the surface since it is brighter than the true surface return. Therefore, we retrack the surface in all data we analyze using a first maximum after maximum gradient tracker. This tracker assumes that the surface return is characterized by a rapid jump in power from the system noise floor to the power of the surface reflection at the two-way travel time

corresponding to the surface. We use the initial CReSIS surface picks as a starting guess and for each trace, search for the maximum power difference between adjacent samples within a +/-15 sample window of the initial guess. Where the initial guess is particularly bad, we tune that window or set some mean guess as the starting point. We filter the results for smoothness, setting any points where the surface jumps more than 10 samples to the value of the previous sample. We then find the first maximum that occurs after our baseline and take this to be the surface. All surface picks were manually reviewed to ensure that there were no significant errors.

Calibration

To invert the radar reflectivities for density, we first absolutely calibrate the received radar power so that it can be compared directly to theoretical calculations. This involves correcting the received power, P_R, for geometric spreading (G), attenuation (A), rough interface scattering loss (L), and some constant radar system offset (S). Equation 3 described the relationship between these variables and the received and calibrated power (P_C) in decibels.

$$\lfloor P_c \rfloor_{dB} = \lfloor P_R \rfloor_{dB} + \lfloor G \rfloor_{dB} + \lfloor A \rfloor_{dB} + \lfloor L \rfloor_{dB} + \lfloor S \rfloor_{dB} \quad (3)$$

We geometrically correct the received power according to equation 4.

$$G = 20 \log_{10} \left[2 \left(h + \frac{d}{1.4} \right) \right] \quad (4)$$

Here h is the aircraft altitude above the surface, d is the depth of the layer, and 1.4 is a reasonable approximation for the mean refractive index of the near surface firn, corresponding to a bulk density of 0.47 gcm^{-3}. The depth of the layer is calculated from the two-way travel time using that same index of refraction. Varying the estimated index of refraction between 1.3 and 1.6 (bulk densities of 0.355 gcm^{-3} and 0.71 gcm^{-3}) results in variations in the geometric correction value of less than 0.05 dB for a typical flight altitude of 500m and a layer at 20 m depth, which is negligible compared with other sources of uncertainty.

In this analysis, we explicitly neglect attenuation. Depth-averaged attenuation rates for the Greenland Ice Sheet are likely to overestimate the rate of near-surface attenuation due to the influence of warm basal ice. It is also difficult to estimate only the near-surface attenuation rate since it is subject to the chemical and seasonally-variable temperature structure as well as the water content of the firn, all of which are difficult to constrain at the spatial scales of this analysis. However, based on a reasonable depth-averaged attenuation rate of 15 dB/km[13], the attenuation correction for a layer at 20 meters depth would increase the apparent power by only 0.6 dB. This is a relatively small correction and would always increase the power of subsurface layers and therefore increase their apparent density. As a result, the maximally conservative approach is to neglect attenuation, rather than introduce additional sources of uncertainty.

The radar system offset correction is typically applied as a single offset to each transect. We first cross-level the data[14] using the minimum network of transects needed to ensure at least three dry snow zone cross-over points for each transect of interest. For each transect, we extract the surface power from our surface picks. For the 2013 and 2017 seasons, we exclude all data points where aircraft roll exceeds 0.05 radians, as this corresponds to the point at which mean surface power falls approximately 1 dB below average due to antenna beam pattern suppression. In 2012, roll data was not reported, so we use the local change in aircraft heading as a proxy for roll and discard all points where the heading gradient exceeds 6e-4 rad/m. We also exclude all points where the 20-point running mean of the surface power is more than one standard deviation outside the 1000-point running mean of the surface power. The objective of this filtering is to remove any unexplained systemic drops in power due to radar receiver problems or transient

surface features in order to retain only the most stable and reliable crossovers for leveling to minimize our uncertainty.

To level the data, we find all crossover points, defined as any pair of traces not from the same transect which fall within 15 m of one another. We take the mean surface power over the radius of the radar footprint around the crossover points and solve for the offset for each transect that minimizes the mean square error between transects over all crossover points. We level the 2017 northwest, 2017 south, and 2013 flight tracks separately and achieve RMS crossover errors after leveling of 1.6 dB, 2.1 dB, and 1.9 dB respectively. This cross-leveling removes relative power offsets between flight transects.

To then determine the absolute radar reflectivity which can be compared with electromagnetic models, we solve for a single absolute calibration constant using firn core cross-overs. Transect 20170328_01 passes within 1 km of the B18 and B26 firn cores, and 20120330_01 passes within 1 km of B26 and B29 firn cores from the 1995 North Greenland Traverse. Density measurements at 1-3 mm depth sampling from 0.2 to 100+ m depth were made on these cores using gamma ray attenuation methods[15–17]. We use these measurements to define the 1D vertical permittivity structure according to equation 1 and use this as input to a radar simulator[18] to model the subsurface reflectivity at each of the crossover points. We then solve for the system offset values that minimize the mean square error between model and observations over a depth range from 15 to 80 m for every trace within 1 km of the crossover point and take the mean value as the true system offset. This method assumes that the firn is largely in steady state below 15 m at these sites and that while the individual layers may not directly match, the seasonal variability has not measurably changed. This is a reasonable assumption, at least in terms of our ability to match the observed radar data[19]. We specifically exclude data less than 15 m deep to minimize the effect of recent changes in firn structure due to new accumulation or water percolation.

Since we have two independent cross-over points for each transect, we can evaluate the stability of this method by comparing the system offsets derived from independent optimization at each crossover. For both flight lines, we find that the difference in estimated system offset from each core site is less than 0.5 dB. A non-trivial source of error in the calibration may be that we do not account for interface roughness in our 1D model, which could result in an overestimation of the system offset. We previously demonstrated by Doppler inversion that subsurface layer roughness at the B26 core is generally less than 0.03 m[19]. Therefore, our calibration constants effectively include a correction for up to 0.03 m of interface roughness. Since these firn cores are located in regions of the ice sheet with the smoothest surface[20] and ice layers may be roughened by ice glands, pipes, and heterogeneous refreezing[21], it is reasonable to think that this correction is an underestimation of interface roughness in our regions of interest and therefore still a conservative calibration approach.

S3. Interpretation of Time Series Data

Interpreting changes in the estimated layer density between 2013 and 2017 is complicated by the fact that changes to either layer thickness or layer density can produce identical changes in layer reflectivity. Since we estimate density from reflectivity, these competing effects are difficult to untangle. For example, an increase in reflectivity, and therefore an inferred increase in estimated density, could plausibly be the result of either layer thinning, layer thickening, layer densification, or some combination thereof, depending on the initial conditions. Fortunately, we can use glaciological constraints to assess the likely modes of change and how they might be reflected in the data. These modes are analyzed below:

	No change in thickness	Layer Thickens	Layer Thins
No change in density	No new refreezing, layer is sufficiently dense in 2013 that minimal compaction occurs under near surface stress regime.	Meltwater refreezes above the 2012 horizon with minimal infiltration into it, minimal compaction.	Physically implausible since compaction thinning is necessarily accompanied by densification, assuming conservation of mass.
	Observable: no change in estimated density	*Observable:* increase or decrease in estimated density	
Density Increases	Meltwater infiltrates and refreezes within the 2012 melt layer.	Meltwater refreezes within and above the 2012 melt layer.	Minimal new refreezing, layer thins and densifies due to compaction and horizontal advection.
	Observable: increase or decrease in estimated density.	*Observable:* increase or decrease in estimate density.	*Observable:* increase or decrease in estimate density.
Density Decreases	Physically implausible scenarios that would require significant englacial melt and runoff or sublimation from within only the 2012 melt layer.		

While we cannot reliably separate the exact mechanism acting on the 2012 melt layer from our time series, we can assess that, where changes are observed, they are best attributed to physical processes which all result in the layer densification, thickening, or both. Therefore, we can reliably assume that spatially consistent changes in the estimated layer density are consistent with continued refreezing at or above the 2012 melt layer between 2013 and 2017.

It is also possible that observed changes might be the result of uncertainty in radar calibration constant or background firn density estimates between seasons. Given that we clearly reproduce no change in density over portions of both flight lines, while showing significant changes in others (see Figure 4), we assess that the changes we observe cannot result from calibration errors since the calibration constant is a single offset to each track and therefore cannot account for intra-flight track variability. Since we use the same model for background firn density for both years and the only change is in the depth of the layer, we assess that this is also unlikely to produce the changes we see since it would require rapid horizontal or vertical gradients in firn density to produce our results.

Supplementary References

1. Karlsson, N. B. *et al.* Ice-penetrating radar survey of the subsurface debris field at Camp Century, Greenland. *Cold Reg. Sci. Technol.* **165**, 1-13 (2019).
2. Machguth, H. *et al.* Greenland meltwater storage in firn limited by near-surface ice formation. *Nat. Clim. Chang.* **6**, 390–393 (2016).
3. MacFerrin, M. J. *et al.* Rapid expansion of Greenland's low-permeability ice slabs. *Nature* **573**, 403–407 (2019).
4. Koenig, L. S. & Montgomery, L. N. Surface Mass Balance and Snow Depth on Sea ice Working Group (SUMup) accumulation on land ice subdataset, Greenland and Antarctica, 1987-2018. *NSF Arctic Data Center*. doi:10.18739/A2ZS2KD0Z (2018).
5. Fettweis, X. *et al.* Reconstructions of the 1900 – 2015 Greenland ice sheet surface mass balance using the regional climate MAR model. *Cryosph.* **11**, 1015–1033 (2017).
6. Fujita, S. *et al.* Nature of radio echo layering in the Antarctic Ice Sheet detected by a two-frequency experiment. *J. Geophys. Res. Solid Earth* **104**, 13013–13024 (1999).
7. Eisen, O., Wilhelms, F., Nixdorf, U. & Miller, H. Revealing the nature of radar reflections in ice: DEP-based FDTD forward modeling. *Geophys. Res. Lett.* **30**, 1-4 (2003).

8. Kovacs, A., Gow, A. J. & Morey, R. M. A reassessment of the in-situ dielectric constant of polar firn. *CRREL Report 93-26* (1993).
9. Kuramoto, T. *et al.* Seasonal variations of snow chemistry at NEEM, Greenland. *Ann. Glaciol.* **52**, 193–200 (2011).
10. Arcone, S. A., Spikes, V. B. & Hamilton, G. S. Phase structure of radar stratigraphic horizons within Antarctic firn. *Ann. Glaciol.* **41**, 10–16 (2005).
11. Medley, B. *et al.* Antarctic firn compaction rates from repeat-track airborne radar data: I. Methods. *Ann. Glaciol.* **56**, 155–166 (2015).
12. Fausto, R. S. *et al.* A snow density dataset for improving surface boundary conditions in Greenland ice sheet firn modeling. *Front. Earth Sci.* **6**, 1–10 (2018).
13. MacGregor, J. A. *et al.* Radar attenuation and temperature within the Greenland Ice Sheet. *J. Geophys. Res. Earth Surf.* **120**, 983–1008 (2015).
14. Chu, W. *et al.* Extensive winter subglacial water storage beneath the Greenland Ice Sheet. *Geophys. Res. Lett.* **43**, 12,484-12,492 (2016).
15. Miller, H. & Schwager, M. Density of ice core ngt37C95.2 from the North Greenland Traverse. *PANGEA.* doi: 10.1594/PANGAEA.57798 (2000).
16. Miller, H. & Schwager, M. Density of ice core ngt42C95.2 from the North Greenland Traverse. *PANGEA.* doi: 10.1594/PANGAEA.57655 (2000).
17. Wilhelms, F. Density of ice core ngt14C93.2 from the North Greenland Traverse. *PANGEA.* doi:10.1594/PANGAEA.56615 (1996).
18. Culberg, R. & Schroeder, D. M. Strong Potential for the Detection of Refrozen Ice Layers in Greenland's Firn By Airborne Radar Sounding. *in IGARSS 2020.* 7033–7036 (2020).
19. Culberg, R. & Schroeder, D. M. Firn Clutter Constraints on the Design and Performance of Orbital Radar Ice Sounders. *IEEE Trans. Geosci. Remote Sens.* 1–18 (2020).
20. Van Der Veen, C. J., Krabill, W. B., Csatho, B. M. & Bolzan, J. F. Surface roughness on the Greenland ice sheet from airborne laser altimetry. *Geophys. Res. Lett.* **25**, 3887–3890 (1998).
21. Brandt, O., Langley, K., Giannopoulos, A., Hamran, S. E. & Kohler, J. Radar response of firn exposed to seasonal percolation, validation using cores and FDTD modeling. *IEEE Trans. Geosci. Remote Sens.* **47**, 2773–2786 (2009).
